# Comparing the diagnostic accuracy, reading time, and inter-rater agreement of breast MRI abbreviated and full protocols: a multi-reader study

**DOI:** 10.1177/02841851231216552

**Published:** 2023-12-19

**Authors:** Roxanna Hellgren, Ernst Tolocka, Ariel Saracco, Brigitte Wilczek, Ann Sundbom, Per Hall, Paul W Dickman

**Affiliations:** 1 Department of Medical Imaging, Division of Breast Imaging, 59570 Södersjukhuset, Stockholm, Sweden; 2 Department of Medical Epidemiology and Biostatistics, 27106 Karolinska Institutet, Stockholm, Sweden; 3 Department of Mammography, 485502 Evidia, Stockholm, Sweden

**Keywords:** Breast, magnetic resonance imaging, neoplasm

## Abstract

**Background:**

Earlier studies have shown that abbreviated protocol magnetic resonance imaging (AB-MRI) has similar diagnostic accuracy as the full protocol (Full MRI).

**Purpose:**

To compare the diagnostic accuracy, reading time, and inter-rater agreement of AB-MRI to Full MRI among women without known increased familial risk of breast cancer or prior biopsy.

**Material and Methods:**

In total, 395 MRI examinations were included in this study. Three readers were blinded to all patient information. The AB-MRI and Full MRI were read separately and in a different random order for each of the readers. Scores 1–2 were considered test negative while scores 3–5 were test positive. A positive reference test was the diagnosis of malignancy; a negative reference test was the absence of a diagnosis of breast cancer within a two-year follow-up. We used a generalized estimating equations approach to compare sensitivity and specificity between the two protocols. We used *t*-tests to compare the average reading time and Krippendorff's alpha to compare inter-rater agreement.

**Results:**

MRI examinations of 395 women (median age=56 years) were evaluated. For AB-MRI and Full MRI, respectively, the sensitivity was 93.0% (95% CI=90.6–95.0) vs. 92.0% (95% CI=89.4–94.1), the specificity was 91.7% (95% CI=90.3–92.9) vs. 94.3% (95% CI=93.2–95.3), average reading time was 67 vs. 126 s, and the inter-rater agreement 0.79 vs. 0.83. The difference in sensitivity was not statistically significant (*P*=0.840), but the difference in specificity was significant (*P*=0.003).

**Conclusion:**

AB-MRI has similar sensitivity, but somewhat lower specificity. The average reading time for the abbreviated protocol is lower, as is inter-rater agreement.

## Introduction

Compared to mammography and ultrasound, dynamic contrast-enhanced magnetic resonance imaging (DCE-MRI) is the most sensitive tool for detection of breast cancer. A meta-analysis of prospective studies comparing DCE-MRI to mammography in women at very high risk found a sensitivity of 0.39 for mammography compared to 0.77 for DCE-MRI (1). The same holds true even among women with average risk. In a study by Kuhl et al. (2), the addition of DCE-MRI to mammography among women of average risk and varying breast density gave an incremental cancer detection rate of 22.6 per 1000 women. In the DENSE Trial, women with the highest mammographic density attending a population-based mammography screening program were selected for an additional MRI examination in the intervention arm. For the women who underwent an MRI, the incremental cancer detection rate was 16.5 per 1000 (3).

One hindrance in implementing MRI as a widespread screening tool is the high cost of equipment and a relatively long image acquisition time. In 2014, Kuhl et al. (4) investigated the value of an abbreviated protocol in a prospective study. The authors defined the abbreviated protocol as the subtracted first post-contrast series and the derived maximum intensity projection (MIP). The results showed a similar diagnostic accuracy for the methods (4). Today, there is no standard definition of an abbreviated protocol, and comparative studies have a wide variety in protocols (5). Generally, all studies conclude that the abbreviated protocol is not inferior to the full protocol in sensitivity. There has been large variation in study populations: women with known increased risk of breast cancer undergoing surveillance (4,6–9); women undergoing preoperative MRI after receiving a biopsy and diagnosis of cancer (10,11); not clearly defined study populations (12–15); or women with a mix of different indication for the MRI examination (16–19).

The value of an abbreviated protocol compared to a full protocol MRI (Full MRI) examination has also been studied in the screening of women of average risk and dense breasts. The first prospective multicenter trial to add an abbreviated MRI (AB-MRI) protocol to mammography in the screening of women of average risk and with dense breasts showed an incremental cancer detection rate of 27.4 per 1000 (20). The incremental cancer detection rate is similar to the results by Kuhl et al. (2) and the DENSE trial (3).

The aim of our multi-reader study was to compare the diagnostic performance, reading time, and inter-rater agreement of breast AB-MRI compared to the Full MRI protocol in a cohort of women without known increased familial risk of breast cancer and regular MR surveillance and where no biopsy was performed before the MRI.

## Material and Methods

### Study participants

This single-institute study was approved by the local institutional review board of our institution, and informed consent was obtained from all participants.

**Fig. 1.**
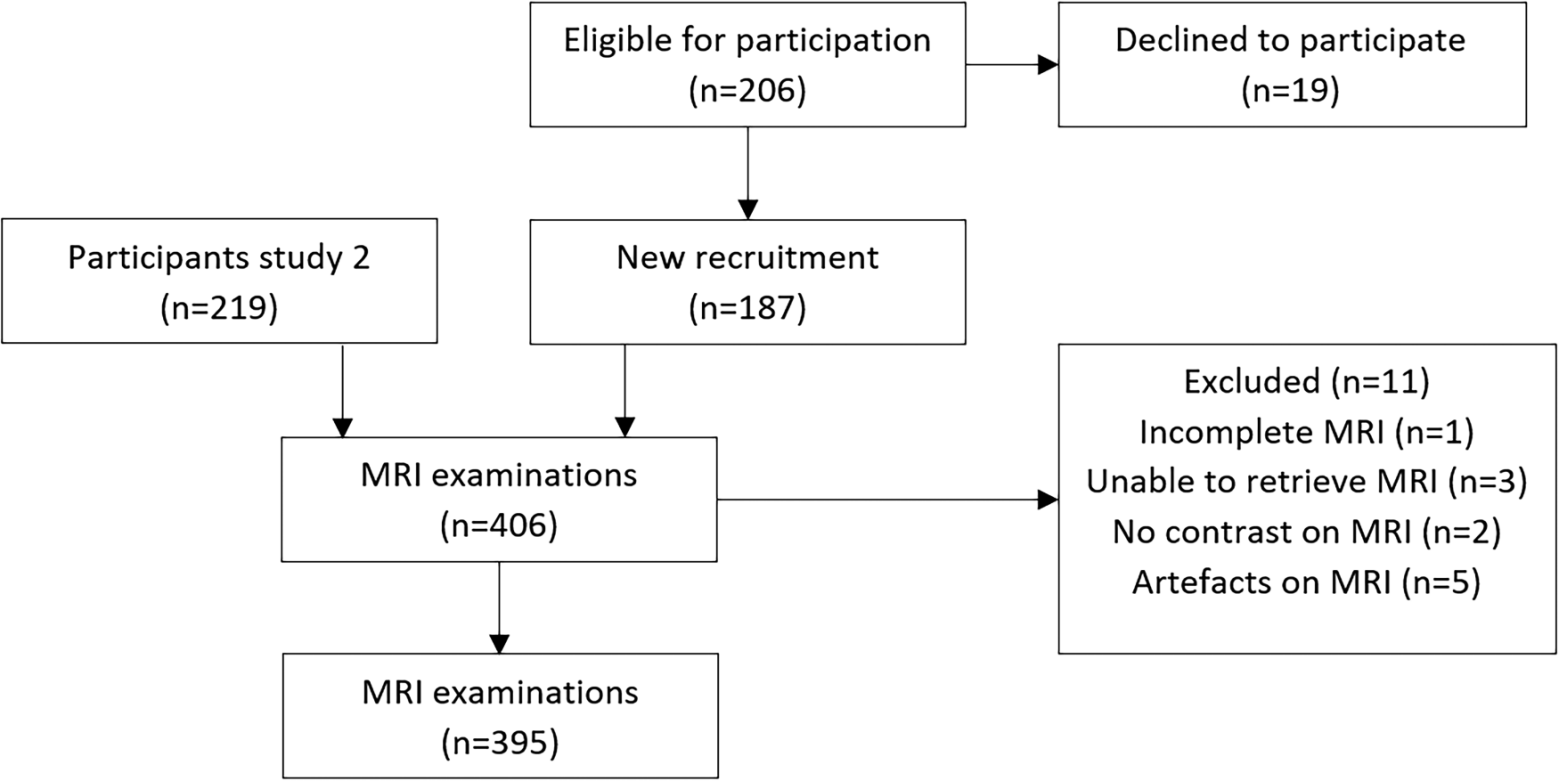
Flow chart of study participants.

A total of 219 women (5 with cancer, 214 without cancer) underwent MRI examinations between January 2015 and August 2016 in a previous study in order to verify the presence of cancer in women with a negative population-based screening mammography and a positive infrared imaging (21). For the purpose of this study, between January 2017 and June 2019, 187 women from the same source population and with a suspicion of breast cancer based on mammography and/or ultrasound were recruited to undergo an MRI examination before biopsy. These women were either recalled after an abnormal screening mammography or referred by a clinician because of a clinical suspicion of breast cancer. Women with fatty involuted breasts, previous breast cancer, previous breast surgery, breast biopsy within the prior six weeks, or ongoing pregnancy were excluded. Women were recruited by radiologists if there were available MRI slots within a few days and rescheduled for biopsy after the MRI examination. Of the 406 women, 11 had to be excluded due to insufficient image quality, leaving 395 women (Fig. 1). None of the study participants had known increased familial risk of breast cancer or prior MRI examinations.

### Index tests and reference standard

Both the AB-MRI and Full MRI protocols were considered index tests. As the reference standard, a pathology diagnosis of malignancy was considered to be true positive and two-year follow-up without a clinical diagnosis of breast cancer was considered to be true negative. We used the final pathology report of surgical specimen for women who underwent primary surgery and the pathology report of biopsies for all other biopsied cases.

### MRI examination

All women underwent a full protocol contrast-enhanced breast MRI examination on the same machine and with the same protocol. MRI was performed according to the guidelines of the European Society of Breast Imaging (22), using a dedicated 16-channel breast coil on a 1.5-T MAGNETOM Aera (Siemens Medical Solutions, Munich, Germany) device. Gadolinium contrast material (Dotarem; Gothia Medical, Billdal, Sweden or Clariscan: GE Healthcare, Boston, USA) was administered intravenously 0.2 mL/kg as a bolus injection with injector followed by 15 mL saline solution.

### Readers and reading methodology

Three radiologists, each with five years of experience in breast MRI and all working full-time at the breast imaging department, read the MRIs. The radiologists were aware of the retrospective nature of the study and that approximately half of the participants were recruited due to a suspicion of cancer and that approximately half had undergone MRI because of a positive infrared imaging score. There was a three-month washout period from the last recruitment to the start of the reading. Patient information was replaced with a study ID before the images were exported to a postprocessing workstation (Syngovia; Siemens). The date of examination was also removed from the image.

Each reader first read the abbreviated protocol and then the full protocol after a one-month washout period. Images were read in a different random order for each reader and each protocol.

### Imaging protocol

Table 1 outlines the full and abbreviated protocols. The abbreviated protocol, with a theoretical scan time of 5 min, was extracted from the full protocol, with a scan time of 30 min. The images were assessed using a postprocessing workstation (Syngovia; Siemens). For the abbreviated protocol, the first post-contrast subtracted image and corresponding MIP were read for scoring. For the full protocol, all series, corresponding subtracted images including the MIP, color map of kinetics, and ADC maps of diffusion-weighted images were available for scoring. No additional information or prior examinations were available to the readers.

**Table 1.** Protocol for Full MRI and AB-MRI.

	Pre-contrast				Post-contrast	
Full MRI	T1W	T2S	STIR	T1W fat-saturated	T1W fat-saturated ×5	DWI
AB-MRI				T1W fat-saturated	First T1W fat-saturated	

AB, abbreviated; DWI, diffusion-weighted imaging; MRI, magnetic resonance imaging; T1W, T1-weighted; T2W, T2-weighted.

### Scoring system

Each breast was scored separately, using the Royal College of Breast Radiologists Breast Group breast imaging classification (23), which is the standard classification system of our country. In this classification system, the following scores are applied: 1 = normal; 2 = benign; 3 = intermediate; 4 = suspicious for malignancy; and 5 = highly suspicious of malignancy. Score 3 is equivalent to BIRADS 4a and requires a biopsy.

Breasts with scores of 1–2 were classified as test negative and breasts with scores of 3–5 were classified as test positive. In breasts with multiple lesions, only the highest score was reported. Note that this system is not BI-RADS.

### Reading time

The time taken for each reader to open a new examination, read the images, and fill in the score was recorded manually by each reader.

### Tumor size

The tumor size is recorded according to the pathology report in all cases except for women who received neoadjuvant chemotherapy. In such cases, the tumor size was measured on the pre-therapeutic MRI.

### Statistical analysis

The sample size was determined to provide 80% power to detect a difference of at least 0.05 between the discordant proportions when using McNemar's test. Expected sensitivity in standard test (Full MRI) was assumed to be at least 86% (4), and it was assumed that the lowest of the discordant proportions was 0.05.

The analyses were conducted on a per-breast basis. We estimated sensitivity and specificity, with exact binomial 95% confidence intervals (CI), for each of the Full MRI and AB-MRI protocols, both aggregated across all readers and separately for each reader (24). We used a generalized estimating equations (GEE) approach with an exchangeable working correlation matrix to estimate and compare sensitivity and specificity between the two modalities while controlling for the fact that multiple readers read the same images (25). We used Krippendorff's alpha (without weighting) to compare agreement between the three readers for each modality (26). We used *t*-tests to compare the average reading time between the two protocols. Stata software version 15.1 was used for statistical analyses.

## Results

### Participant characteristics

A total of 395 women (median age = 56 years; age range = 40–74 years) constituted the study population. A total of 178 women had breast cancer, 131 women were recalled from screening because of a suspicious finding on the mammogram, and 47 women were referred to our clinic due to clinical symptoms. The main characteristics with regard to malignancies are presented in Table 2.

**Table 2.** Characteristics of study participants.

	Participants (n)
Total no. of participants	395
Diagnosis of breast malignancy	178
Unilateral malignancy	165
Bilateral malignancy	13
Mammography screening detected breast malignancy	131
Clinically detected breast malignancy	47
Neoadjuvant chemotherapy	25

### Lesion characteristics

Table 3 describes lesion characteristics according to pathology. There were 178 women with cancer, of whom 13 had bilateral cancer. This yielded 191 breasts with cancer. We also found 29 non-malignant results of biopsies.

**Table 3.** Breast findings according to pathology.

Description of reported results	No. of findings
No. of women with malignancy	178
No. of women with bilateral malignancy	13
No. of breasts with malignancy	191
No. of breasts with ductal cancer in situ without invasive component	16
No. of breasts with invasive tumor	175
Type of invasive tumor	
NST	141 (80)
Lobular	31 (18)
Cribriform	2 (1)
Papillary	1 (1)
Grade of invasive tumor according to Nottingham	
1	45 (26)
2	87 (50)
3	43 (24)
No. of non-malignant biopsied findings in the breasts	29
Adenosis	6
Borderline phyllodes	1
Atypical ductal hyperplasia	2
Hyperplasia without atypia	2
Cystic fibroadenosis	2
Fibroadenoma	7
Columnar cell hyperplasia	1
Columnar cell hyperplasia with atypia	1
Radial scar	3
Papillomatosis	1
Fibrosis	1
Papilloma	2

Values are given as n or n (%).

NST, no special type.

### Size of malignancies

For invasive tumors, the median size was 16 mm (range = 3–150 mm). For multifocal tumors, the size of the largest invasive component was reported. For ductal carcinoma in situ without invasive component, the median size was 35 mm (range = 7–100 mm).

### Diagnostic accuracy for abbreviated and full protocol

Analysis was carried out on a per-breast basis, so that there was a total of 790 breasts in the analysis. Estimates for sensitivity, specificity, and receiver operating characteristic (ROC) for each protocol, both aggregated across all readers and separately for each of the three readers, are presented in Table 4. The overall sensitivity was 93.0% for AB-MRI and 92.0% for Full MRI. The difference between the two was not statistically significantly different based on the GEE analysis (*P* = 0.51). The overall specificity was 91.7% for AB-MRI and 94.3% for Full MRI. The difference between the two was statistically significant based on the GEE analysis (*P* = 0.003).

**Table 4.** Sensitivity, specificity, and ROC for each protocol.

	Sensitivity (%)	Specificity (%)	ROC
*Aggregated*			
AB-MRI	93.0 (90.6–95.0)	91.7 (90.3–92.9)	0.92 (0.91–0.94)
Full MRI	92.0 (89.4–94.1)	94.3 (93.2–95.3)	0.93 (0.92–0.94)
*Reader 1*			
AB-MRI	94.8 (90.6–97.5)	93.8 (91.6–95.6)	0.94 (0.92–0.96)
Full MRI	95.3 (91.2–97.8)	94.8 (92.7–96.5)	0.95 (0.93–0.97)
*Reader 2*			
AB-MRI	88.5 (83.1–92.6)	92.0 (89.5–94.0)	0.90 (0.88–0.93)
Full MRI	86.4 (80.7–90.9)	94.3 (92.2–96.0)	0.90 (0.88–0.93)
*Reader 3*			
AB-MRI	95.8 (91.9–98.2)	89.3 (86.6–91.7)	0.93 (0.91–0.94)
Full MRI	94.2 (89.9–97.1)	93.8 (91.6–95.6)	0.94 (0.92–0.96)

Values in parentheses are 95% CI.

AB-MRI, abbreviated MRI protocol; CI, confidence interval; Full MRI, full MRI protocol; ROC, receiver operating characteristic.

Even though the specificity was somewhat lower for the abbreviated protocol, there were similar ROC values for the two protocols, with an aggregated ROC value of 0.92 for AB-MRI and 0.93 for Full MRI.

### Inter-rater agreement

Krippendorff’s alpha, a measure of inter-rater agreement, was 0.79 (95% CI = 0.75–0.84) for AB-MRI and 0.83 (95% CI = 0.78–0.87) for Full MRI.

### Reading time

The mean reading time across all readers was 67 s (95% CI = 64.5–68.7) for AB-MRI and 126 s (95% CI = 123–130) for Full MRI. The difference was statistically significant, with *P* <0.0005.

## Discussion

In this multi-reader blinded study, we aimed to compare the diagnostic accuracy, reading time, and inter-rater agreement of AB-MRI and Full MRI for the detection of breast cancer among 395 women. For AB-MRI versus Full MRI, the aggregated sensitivity was 0.93 and 0.92 and the difference was not statistically significant (*P* = 0.51). The specificity was 0.92 and 0.94 and the difference was statistically significant (*P* = 0.003). There was a significant difference in mean reading time of 67 s and 126 s, respectively (*P* = 0.000). The inter-rater agreement was 0.79 and 0.83, respectively.

Most studies have shown a similar sensitivity and specificity between the methods (4,7,9,13–15,17,19,27). Our results confirm similar sensitivity, but not similar specificity. As in studies by Chen et al., our results show lower specificity for the abbreviated protocol (12,28). Our explanation is that the detection of contrast-enhancing findings can be achieved with one pre- and one post-contrast acquisition, but since we chose the minimum of the abbreviated protocol, with only access to the subtracted pre- and post-contrast image and the derived MIP, we had no additional information for further characterization.

The reported reading time of earlier studies is in the range of 28–179 s for AB-MRI (4,7,8,11,12,17). The mean reading time was 28 s (4), 37 s (12), 44 s (11) 93 s (8), 120 s (17), and 179 s (7). In our study, the mean reading time was 67 s, which seems to be reasonably somewhere in the middle of earlier results. Our explanation for the short reading time both for AB-MRI and Full MRI is the fact that in a study setting, the radiologists only needed to make a score without any repercussions for the patient such as missing a cancer, initiating false-positive recalls and biopsies, or causing an unnecessary mastectomy. Although the observed reading time is not equivalent to the true reading time in a clinical setting, we can still conclude that there is a reduction of reading time when there are less images to interpret.

We were able to identify two studies that have reported inter-rater agreement between two experienced readers. Machida et al. found the inter-rater agreement to be 0.56 for AB-MRI and 0.69 for Full MRI (13) while Oldrini et al. reported 0.80 and 0.91, respectively (18). Similar to Oldrini et al., we found good agreement between readers with 0.79 and 0.83 respectively (18).

The main aim and strength of our study is that biopsies were performed after the MRI examination. The images contained no information that could help the readers identify breasts with cancer and the readers had no clinical information or prior examinations in decision making. Our study participants were women without known increased familial risk of breast cancer and regular MRI surveillance and approximately three-quarters of women with malignancy (131 of 178) were recalled due to a suspicious finding on the screening mammogram. In the national screening program, mammography is performed every other year.

The present study has some limitations. One of them is that the readers could have remembered the cases from earlier clinical care. A similar limitation in the study design was the short washout period between reading the AB-MRI and Full MRI protocols. However, since AB-MRI is the inferior method with less information, and all the abbreviated examinations were scored first, we believe that the relatively short washout period did not influence the results. Second, similarity in the level of experience among readers is another factor that may decrease the generalizability of the results. All three readers had five years of experience in breast MRI and longer experience in breast diagnostics, working in a breast-dedicated unit. The readers were neither super experts in breast MRI nor novices. Most likely, they represent an average level of experience in the profession. A third limitation of our study is that the cohort was enriched with cancers already detected by mammography or a clinical finding, which does not reflect a normal screening setting. This knowledge may have influenced the readers’ assessment. Our study is not unique in this regard, and approximately half of the published studies on this topic have a cancer-enriched cohort (7,10,11,13–15,17–19,29–31). There were relatively few lesions with benign histology, most likely because of exclusion of all women with prior breast surgery. We also lack data for cancers only detected by MRI.

In a screening setting, both the accuracy of the method as well as cost-effectiveness need to be considered. The AB-MRI protocol reduces acquisition time and reading time while maintaining a high diagnostic accuracy. Although the time needed for intravenous cannulation, positioning the patient on the MRI table, and proper positioning of the breasts in the coil cannot be influenced, we still believe that the AB-MRI protocol will reduce the total cost since the total scan time will be shortened. This improvement in cost-effectiveness may be necessary when recommendations for screening with MRI are expanded to include women without family history of breast cancer, but with extremely dense breasts (32) and women with a lifetime risk of breast cancer >20% (33).

In conclusion, compared to the Full MRI protocol, the AB-MRI protocol reduces acquisition and reading time while maintaining a similar sensitivity and good inter-rater agreement. The protocol potentially decreases the total examination cost, which is necessary for a more widespread use of MRI in breast cancer screening.
